# The outcome of Kasai portoenterostomy after day 70 of life

**DOI:** 10.3389/fped.2022.1015806

**Published:** 2022-10-21

**Authors:** Fangran Liu, Fanny Yeung, Patrick Ho Yu Chung

**Affiliations:** Division of Paediatric Surgery, Department of Surgery, School of Clinical Medicine, Li Ka Shing Faculty of Medicine, The University of Hong Kong, China

**Keywords:** biliary atresia, Kasai portoenterostomy, liver transplant, portal hypertension, cholangitis

## Abstract

**Background:**

The age at Kasai portoenterostomy (KPE) was reported to correlate with the prognosis of patients with biliary atresia (BA) and that a late KPE is bounded to be failure. Herewith, we reported the outcome of patients receiving KPE after day 70 of life. In addition, the prognostic indicators were evaluated.

**Materials and Methods:**

This was a retrospective analysis and all BA patients receiving KPE after day 70 of life in a tertiary centre between 1980 and 2018 were evaluated.

**Results:**

A total of 164 KPE procedures were performed during the study period and 62 cases were done after day 70 of life which were included in this study. The median follow up period of these patients was 10.6 years (range: 4.5 to 41.5 years). Thirty-nine patients (62.9%) patients were able to achieve jaundice clearance at 6 months after KPE. The NLS rate was 53.2% (*n* = 33) as recorded at the time of writing. There was no statistical difference in the age at KPE between native liver survivors and patients requiring liver transplant. For complications among the native liver survivors (*n* = 33), portal hypertension and recurrent cholangitis were found in 63.6% and 30.3% of these patients. There was also no significant difference in the age at KPE between those who developed portal hypertension and recurrent cholangitis (*p* = 0.451 and *p* = 0.173 respectively). Regarding the prognostic indicators in predicting NLS, pre-KPE bilirubin, alkaline phosphatase (ALP) and gamma-glutamyl transferase (GGT) were significantly higher among patients requiring liver transplant (*p* = 0.012, =0.011 and =0.017 respectively). The bilirubin level at 6 months after KPE was also higher among patients who required liver transplant (*p* = 0.016).

**Conclusion:**

More than half of the BA patients can survive for 10 years with their native liver despite KPE was performed after day 70 of life. However, they have a higher chance to develop BA-related complications. The level of pre-KPE bilirubin and ductal enzymes as well as post-KPE bilirubin are prognostic indicators to predict NLS.

## Introduction

Biliary atresia (BA) is a congenital devastating disease and etiology remains largely unknown (1, 2). Even though BA is rare, a vast majority of burden is imposed on the patients, their families and the community (3). Kasai portoenterostomy (KPE) is the widely accepted surgical treatment for BA. Not uncommonly, KPE could be delayed due to various reasons such as the failure to recognize jaundice and the passage of acholic stool as features of BA (4). While the best timing for KPE remains unclear, most published studies have suggested that the operative outcome is linked to the age of KPE and the later the operation, the worst is the outcome (5, 6). In this study, we reported the outcome of a specific group of BA patients who had received KPE after day 70 of life. Moreover, we would like to analyze if there are prognostic indicators that may predict the native liver survival (NLS) rates when KPE is performed at this age.

## Materials and methods

This was a retrospective study conducted in a tertiary referral centre with 50 years of experience in treating BA. Medical records of patients who underwent KPE from 1980 to 2018 were retrieved and KPE performed after day 70 of life were included for analysis. In all cases, the diagnosis of BA was confirmed by surgical exploration to reveal an atretic bile duct with or without operative cholangiogram. KPE was performed according to the conventional open approach at the time when the diagnosis of BA had been established. During the study period, all KPE procedures were performed by 5 surgeons with the same surgical principle across different generations. Since 2004, a 6-week course of oral steroid therapy (4 mg/kg/day with dosage cut half every two weeks) was given to patients after KPE. Patients were excluded from the analysis if their follow up data were incomplete. The primary outcome was NLS rate which is defined as survival with own liver and has not been listed for liver transplant. Secondary outcomes included the development of BA-related complications (7), including portal hypertension (defined as the presence of oesophago-gastric varix and/or hypersplenism) and recurrent cholangitis (an episode of cholangitis was defined by the presence of fever [core body temperature >38.5 degree Celsius] and elevated serum bilirubin level above 20 µmol/L on two consecutive blood samples requiring intravenous antibiotics). Potential prognostic indicators that were analyzed included (1) the age at KPE; (2) serum bilirubin and ductal enzymes before KPE and (3) Post-KPE bilirubin at 1-,3- and 6-month intervals (8–12).

Statistical analysis was performed with IBM SPSS Statistics for Windows, version 23 (IBM Corp., Armonk, N.Y., USA). Continuous variables were reported as mean ±SD or median (range) when appropriate. Categorical variables were compared with chi-square test. NLS was estimated with Kaplan-Meier analysis. For all analyses, a *p* value <=0.05 was considered to be statistically significant. This study has been approved by the Institutional Review Board of the participated centre and was performed according to the Declaration of Helsinki (IRB number: UW20-156).

## Results

**Figure 1:**
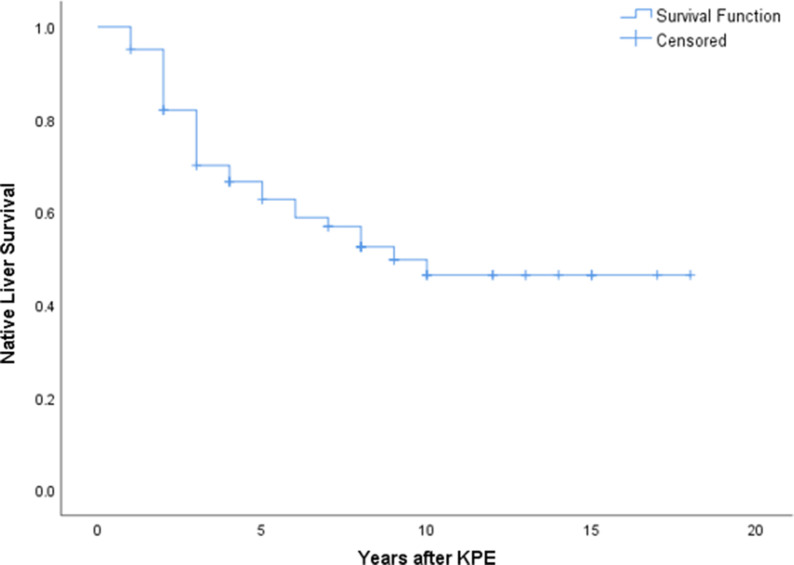
Kaplan Meier analysis revealed approximately 50% of patients with KPE after day 70 of life could achieve long term native liver survival.

**Figure 2:**
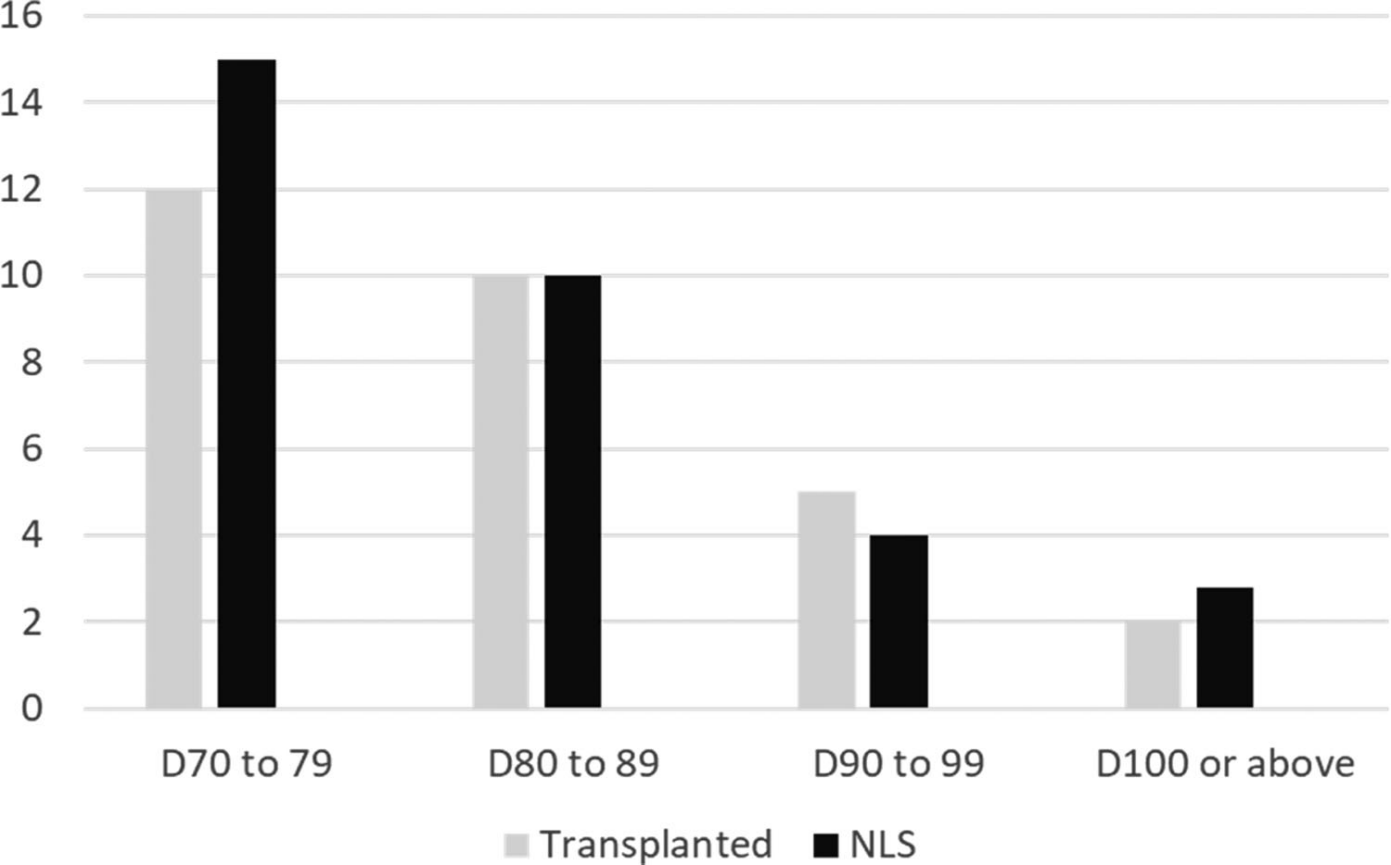
Comparison of native liver survivors and transplanted patients in different age groups showed a progressive increase in the proportion of patients requiring LT as the age at KPE advanced. (x axis: age at KPE; y axis: number of patient).

A total of 181 patients underwent KPE during the study period. Seventeen patients were excluded due to incomplete data. There were an additional of 12 patients receiving primary liver transplant that were not included in this study. Among 164 KPE procedures, 62 (37.8%) were performed after day 70 of life and included for analysis. There were 42 female patients and their mean age at KPE was 82.6 ± 10.4 (day of life). Five patients suffered from syndromic association. Thirty-six patients received post-KPE steroid therapy. The median follow up period was 10.6 years (range: 4.5 to 41.5 years). At 6 months after KPE, thirty-nine patients (62.9%) were able to achieve jaundice clearance (total serum bilirubin below 20 µmol/L). At the time of writing, 33 patients (53.2%) were still living with their native liver and not requiring liver transplant listing. On the other hand, patients who received KPE before day 70 of life (*n* = 102), their 6-month jaundice clearance rate was 68.6% (*n* = 70) and NLS rate was 65.7% (*n* = 67). Using Kaplan Meier analysis to estimate the long term survival of patients receiving KPE after day 70 of life, it was revealed that approximately 50% of them could continue to survive with their own liver in 10 years (Figure 1). The mean age at LT was 21.5 ± 5.4 months. There was no significant difference in the mean age at KPE between native liver survivors and transplanted patients [82.1 ± 10.2 vs. 83.1 ± 9.1 (day of life), *p* = 0.382]. Next, we performed a sub-group analysis to evaluate the impact of age at KPE on the NLS. The age at KPE was divided into four groups and the percentage of patients requiring liver transplant was 44.4% for KPE at day 70–79 of life; 50% for KPE at day 80–89 of life; 55.5% for KPE at day 90–99 of life and 40% for KPE on or after day 100 of life, *p* = 0.866 (Figure 2).

Regarding potential prognostic indicators, we observed the pre-KPE bilirubin was lower in the native liver survivors when compared to transplanted patients (100.3 ± 23.1 µmol/L vs. 139.9 ± 37.4 µmol/L, *p* = 0.012). In addition, the levels of ductal enzyme were also significantly lower in the native liver survivors (ALP: 366.3 ± 154.3 vs. 485.1 ± 291.3 [U/L], *p* = 0.011; GGT: 466.2 ± 195.4 vs. 538.9 ± 375.6 [U/L], *p* = 0.017). Post-operatively, there were no significant differences observed in the serum bilirubin level at 1- and 3-month after KPE between the two groups (1 month: 62.7 ± 44.6 vs. 88.1 ± 53.9, *p* = 0.291; 3-month: 38.9 ± 33.9 vs. 54.0 ± 47.5, *p* = 0.096). However, native liver survivors had a significantly lower serum bilirubin level at 6 months after KPE (29.6 ± 21.7 µmol/L vs. 47.0 ± 33.5 µmol/L, *p* = 0.016) (Table 1).

**Table 1:** Comparison of peri-operative liver function between native liver survivors and transplanted patients who underwent KPE after day 70 of life.

	Native Liver Survivors (*n* = 33)	Patients Transplanted (*n* = 29)	*p* value
Mean age at Kasai (day of life)	82.1 ± 10.2	83.1 ± 9.1	0.382
Mean value for pre-KPE bilirubin (µmol/L)	100.3 ± 23.1	139.9 ± 37.4	***0*** *.* ***012***
Mean value for pre-KPE ALP (U/L)	***366.3 ***± ***154.3***	***485.1 ***± ***291.3***	***0***.***011***
Mean value for pre-KPE GGT (U/L)	***466.2 ***± ***195.4***	***538.9 ***± ***375.6***	***0***.***017***
Mean value for post-KPE bilirubin at 1 month (µmol/L)	62.7 ± 44.6	88.1 ± 53.9	0.291
Mean value for post-KPE bilirubin at 3 months (µmol/L)	38.9 ± 33.9	54.0 ± 47.5	0.096
Mean value for post-KPE bilirubin at 6 months (µmol/L)	*29.6 *± *21.7*	*47.0 *± *33.5*	***0***.***016***

Next, we evaluated portal hypertension and recurrent cholangitis among native liver survivors (*n* = 33). For portal hypertension, 21 patients suffered from this complication which accounted for 63.6% of all native liver survivors. Ten patients (30.3%) had experienced recurrent cholangitis. The age at KPE did not differ between patients with and without these two complications (portal hypertension: 83.3 ± 9.2 vs. 81.2 ± 12.3 [day of life] *p* = 0.451; recurrent cholangitis: 84.1 ± 14.5 vs. 80.3 ± 8.4 [day of life], *p* = 0.173 (Table 2). However, the incidence for both complications was higher than patients who received KPE before day 70 of life Portal hypertension (PHT) and recurrent cholangitis were found in 51.6% and 27.5% of them respectively.

**Table 2:** The mean age at KPE for native liver survivors who suffered from portal hypertension and recurrent cholangitis.

	Portal hypertension +ve (*n* = 21)	Portal hypertension –ve (*n* = 12)	*p* value
Mean Age at KPE (days)	83.3 ± 9.2	81.2 ± 12.3	0.451
	Recurrent cholangitis +ve (*n* = 10)	Recurrent cholangitis –ve (*n* = 23)	
Mean Age at KPE (days)	84.1 ± 14.5	80.3 ± 8.4	0.173

## Discussion

Since the introduction of KPE as the curative treatment for BA, factors affecting the operative outcomes have been studied extensively. The age at KPE has drawn the most attention from researchers and it is generally believed that the timing of operation correlates with the operative outcome (13–16). Early KPE is recommended to avoid the presence of irreversible liver injury due to disease progression. It has been shown that most BA patients generally survive with their native liver for a long period if they undergone KPE before 90 days (17). According to a study before, if KPE can be done before 75 days of age, more than 40% of liver transplantations could be spared (18). Nonetheless, there were also studies reporting satisfactory outcome in an apparently “late” KPE. Controversy exists at whether KPE should be offered to late presenters or these patients should be offered primary liver transplant, due to the higher failure rate (6). Nevertheless, proponents for late KPE concerned about the risk and complications associated with liver transplant, in addition to the scarcity of donor in many places. Our previous report has revealed that that KPE before day 70 of life was associated with a higher chance of successful drainage (19). Therefore, we herewith use day 70 of life as the cut off to evaluate the outcome of a “late” KPE.

Our findings suggested that patients who underwent KPE after day 70 of life still had more than 50% of chance to survive with their native liver for a considerable period of time. This percentage appeared to be higher than one of the earliest studies on “late” KPE by Chardot et al. in 2001 (20). More recently, Uecker et al. also reported the outcome of 11 patients receiving KPE at a mean age of 108 days. A high transplant rate (73%) was observed but the average age at transplant was almost 1.7 years. The authors advocated that KPE should still be the preferred primary treatment option (21). When KPE can successfully restore biliary drainage, liver transplant can be avoided or at least be deferred to an older age when the risk is lower. Even though the technique of liver transplantation is matured in well-established centres, this operation still carries significant risk of morbidity as well as mortality. KPE can also serve as a bridging therapy particular for places where organ supply is in shortage. Transplant recipients have to use life-long immunosuppressant which predisposes them to an immune-deficient status. In the worst case, haematological malignancy can develop (2, 19). The side-effects of immunosuppressant are more prominent in younger patients and hence early liver transplant should be avoided as far as possible. With more than 50% long-term NLS, we concur that KPE should remain the primary treatment even for late presenters.

Our results have shed the light on the feasibility to salvage late presenter by KPE. Indeed, our findings here were similar to a recent study by Khayat et al. that also reported 56% NLS rate among patients with KPE done at a median age of day 77 of life (22). Nevertheless, we admitted that this is over-simplified to recommend that KPE should be offered to all late presenters without precautions. Therefore, in addition to existing knowledge, we attempted to identify prognostic factors that could potentially predict a lower chance of successful operation when it is done after day 70 of life. With this regard, we performed a sub-group analysis to investigate whether the age at KPE would impact on the outcome by dividing them into different age groups. We observed a trend of lower NLS rate as the age at KPE advanced (Figure 2). This is also in line with the general belief that more extensive and irreversible liver damage occurs as the disease is allowed to progress. Although a slight but non-significant improvement was noted in the most advanced group, we believed this could be related to patient selection as some patients with presentation beyond day 100 of life had undergone primary liver transplant and excluded.

It is still unclear from the literature what are the prognostic markers to predict the outcome of patients who received KPE after day 70 of life. Here, we revealed that the pre-KPE serum bilirubin and ductal enzyme levels were significantly lower in NLS than transplanted patients. We chose bilirubin and ductal enzyme to evaluate as these two markers are reflective of the underlying biliary obstruction. This indicated that the severity of biliary obstruction before the operation had a detrimental effect on the operative outcomes. If the obstruction is too severe, the chance of successful post-KPE drainage could be jeopardized. Post-operatively, the serum bilirubin level at 6 months could be outcome-determining. This finding is also supported by previous studies reporting the prognostic value of early post-KPE bilirubin level. Of note, there were no significant difference in the level during the first three months after KPE. Therefore, the prognosis of the patients need not to be determined soon after KPE. On the other hand, by knowing the predictive factors ahead, clinicians can formulate the next management plan in advance to minimize morbidity (23, 24). For patients with poor prognostic indicators, they can be prepared for liver transplant with adequate time. It is always beneficial if the work up can be completed in time especially in places where organ supply from deceased donor is limited. This could also facilitate an early identification of potential living donor. In addition to biochemical index, when we compared the proportion of NLS according to the age at KPE, we noticed a higher proportion of patients would require liver transplant as the age at KPE advanced.

Besides the evaluation of NLS, we were also interested to examine the impact of late KPE on the future development of complications. Portal hypertension and cholangitis are two common BA complications and it is estimated that 50% of BA native liver survivors will suffer from one or both of these (25, 26). In this cohort, the prevalence was slightly higher with approximately two-third of patients suffering disease-related complications. However, we could not detect a significant difference in the age at KPE between patients with and without these complications. This could be related to the small sample size as 33 native liver survivors were included for analysis. Nevertheless, the incidence of both complications was higher than the overall figures as reported in our previous study (PHT: 51.6% and recurrent cholangitis: 27.5%) (19). The findings have enlightened us about the impact of late KPE in the subsequent development of complications. Thus, in addition to a better NLS, we recommend that KPE should be performed before day 70 of life whenever possible to avoid long term complications. Patients who undergo a late KPE should be closely monitored for portal hypertension and cholangitis. Surveillance imaging and endoscopy should be carried out to detect portal hypertension which can be clinically silent but life-threatening. The onset of BA complications can potentially exacerbate the hepatic injury and subject the patient to liver failure.

We acknowledge there are several limitations with this study. As we focused on patients with late KPE, the sample size has been further reduced for this rare disease. In addition, this study is subjected to the bias associated with a retrospective design. The study period was over 40 years and there were inevitably changes in the management details such as operative technique and the use of adjuvant medications. We admitted that this could affect the analysis but the variations in practice have been made minimized by limiting the number of in-charge surgeons who all followed the same surgical principle. The diagnosis of portal hypertension was based on clinical evidence and direct portal measurement has not been carried out. The true prevalence of portal hypertension might therefore be under-estimated as early asymptomatic patients were not diagnosed.

Despite the aforementioned study, we would like to conclude that patients who undergo KPE after day 70 of life are expected to have 50% native liver survival at 10 years follow up. However, early KPE is still preferred as late operation predisposes patients to a higher risk of developing BA related complications. Pre-KPE bilirubin and ductal enzyme levels predict the outcomes of KPE. Post-operatively, the bilirubin at 6 months indicates the prognosis of the patient.

## Data Availability

The raw data supporting the conclusions of this article will be made available by the authors, without undue reservation.
